# Chemical and behavioral integration of army ant-associated rove beetles – a comparison between specialists and generalists

**DOI:** 10.1186/s12983-018-0249-x

**Published:** 2018-03-16

**Authors:** Christoph von Beeren, Adrian Brückner, Munetoshi Maruyama, Griffin Burke, Jana Wieschollek, Daniel J. C. Kronauer

**Affiliations:** 1 Ecological Networks, Department of Biology, Technische Universität Darmstadt, 64287 Darmstadt, Germany; 2 Laboratory of Social Evolution and Behavior, The Rockefeller University, New York, NY 10065 USA; 3 The Kyushu University Museum, Hakozaki 6-10-1, Fukuoka, 812-8581 Japan; 4 Bard College, Annandale-on-Hudson, NY 12504 USA

**Keywords:** Chemical mimicry, Myrmecophile, Army ant, Host-parasite interaction, Social integration, Cuticular hydrocarbons, DNA barcoding, *Eciton burchellii foreli*, *Ecitophya*, *Ecitomorpha*, *Tetradonia*

## Abstract

Host-symbiont interactions are embedded in ecological communities and range from unspecific to highly specific relationships. Army ants and their arthropod guests represent a fascinating example of species-rich host-symbiont associations where host specificity ranges across the entire generalist - specialist continuum. In the present study, we compared the behavioral and chemical integration mechanisms of two extremes of the generalist - specialist continuum: generalist ant-predators in the genus *Tetradonia* (Staphylinidae: Aleocharinae: Athetini), and specialist ant-mimics in the genera *Ecitomorpha* and *Ecitophya* (Staphylinidae: Aleocharinae: Ecitocharini). Similar to a previous study of *Tetradonia* beetles, we combined DNA barcoding with morphological studies to define species boundaries in ant-mimicking beetles. This approach found four ant-mimicking species at our study site at La Selva Biological Station in Costa Rica. Community sampling of *Eciton* army ant parasites revealed that ant-mimicking beetles were perfect host specialists, each beetle species being associated with a single *Eciton* species. These specialists were seamlessly integrated into the host colony, while generalists avoided physical contact to host ants in behavioral assays. Analysis of the ants’ nestmate recognition cues, i.e. cuticular hydrocarbons (CHCs), showed close similarity in CHC composition and CHC concentration between specialists and *Eciton burchellii foreli* host ants. On the contrary, the chemical profiles of generalists matched host profiles less well, indicating that high accuracy in chemical host resemblance is only accomplished by socially integrated species. Considering the interplay between behavior, morphology, and cuticular chemistry, specialists but not generalists have cracked the ants’ social code with respect to various sensory modalities. Our results support the long-standing idea that the evolution of host-specialization in parasites is a trade-off between the range of potential host species and the level of specialization on any particular host.

**Electronic supplementary material:**

The online version of this article (10.1186/s12983-018-0249-x) contains supplementary material, which is available to authorized users.

## Background

*„Die gelungenste Anpassungstactik ist aber jedenfalls die, dem übermächtigen Gegner als Freund sich anzuschließen und den Grundsatz zu befolgen: ‚Mit den Wölfen muss man heulen’. Wem das gelingt, dem ist eben durch die Gesellschaft seiner furchtbarsten Feinde ein mächtiger Schutz und eine reichgedeckte Tafel gesichert.“* Erich Wasmann (1895)

*It is the most successful adaptive strategy to join the superior opponent as a friend and to follow the principle: To run with the pack. For those who succeed, the companionship with their most terrifying enemies guarantees powerful protection and richly laden tables.*

In 1894, the Austrian entomologist Erich Wasmann provided a first inventory of ant-associated arthropods, in which he listed more than 1000 species [1]. His seminal work formed the foundation for the study of myrmecophiles (ant lovers) [2–5]. Current estimates of myrmecophile diversity range from 10,000 to 100,000 species [6, 7]. This species richness is mirrored by a great diversity of life styles spanning from opportunistic ant predators to symbionts that mandatorily depend on the association with ants [2, 8, 9].

Host-symbiont interactions are embedded in larger ecological networks and range from rather general to highly specific relationships. How these interactions evolve as symbionts become more and more host-specific is an important question in evolutionary biology in general and parasitology in particular [10]. Host specificity in ant-myrmecophile interactions is known to fall along a generalist - specialist continuum [11]. Even though data about host preferences are scarce (for notable exceptions see e.g. [12–14]), collection records indicate that increasing host specificity is often accompanied by increasing morphological, physiological, and behavioral specialization [2–4, 8, 11, 13].

The aim of the present study was to compare the level of specialization to myrmecophily between ant-associated beetle generalists and beetle specialists. Generalist myrmecophiles with a broad host spectrum often lack conspicuous adaptations to the life with ants and rather resemble free-living relatives [2–4, 7, 8]. For instance, most myrmecophiles of red wood ants occupy a broad host spectrum and are morphologically generalized [15, 16]. Interestingly, the majority of these myrmecophiles do not mimic the host’s chemical recognition cues [17], an otherwise common strategy among myrmecophiles to evade host detection [18, 19]. In the last years, we systematically assessed the host specificity of army ant myrmecophiles at La Selva Biological Station, Costa Rica [20–23]. Common generalist army ant associates are beetles of the genus *Tetradonia* (Staphylinidae: Aleocharinae: Athetini) [24], and those are the generalists we studied here. They prey upon ant workers of several host species (range 2–6 host ant species) [20] and show no apparent anatomical modifications compared to free-living staphylinids [25–27]. They are not well integrated into the host colony, where they can usually be found at the periphery, commonly in ant refuse deposits [5, 20, 24].

On the other hand, host specific myrmecophiles often possess conspicuous adaptations to myrmecophily including behavioral, acoustical, anatomical or chemical traits [7, 18, 19, 28–30]. Striking specialist army ant associates are beetles of the tribe Ecitocharini (Staphylinidae: Aleocharinae) [31], and those are the specialists we studied here. Host records indicate that all members of this tribe (10 genera with approximately 20 species [32]) are specifically associated with a single army ant species [11, 25, 27, 32, 33]. These kleptoparasites feed on the prey of host ants while living inside the colonies of army ants [25], in which they are extremely well integrated, behaving as if they were colony members [24, 31]. Most strikingly, some members of this beetle tribe have undergone exceptional anatomical modifications by resembling the body shape and sometimes even the coloration of host ants [25, 27, 32], a type of mimicry termed Wasmannian mimicry [34]. There is no doubt that Wasmannian mimics experienced strong selective pressures to evolve the ant-like habitus, because a myrmecoid body shape has evolved at least 12 times independently within the staphylinid subfamily Aleocharinae [25]. Since army ants have generally poor vision [31, 35, 36], host resemblance in color likely represents an adaptive response to vertebrate predators such as army ant-associated birds [2, 31]. The selective agent driving these beetles towards body shape resemblance is, however, still unknown [25, 31, 32]. Many Wasmannian mimics parasitize army ants with a subterranean life style and differ in body coloration from host ants (see [4]), indicating that visual predators cannot be the only selection pressure acting on Wasmannian mimics. Erich Wasmann himself suggested that tactile inspection of host ants could be responsible for the independent evolution of this ant-like habitus, and called this phenomenon tactile mimicry [37].

In the present study, we compared the behavioral and chemical integration mechanisms of *Tetradonia* beetles and Wasmannian mimics, representing two extremes of the generalist – specialist continuum, respectively. Similar to a previous study of *Tetradonia* beetles [20], we combined DNA barcoding with morphological studies to define species boundaries in ant-mimicking beetles, which allowed us, in combination with community sampling of *Eciton* army ant colonies, to determine host specificities in great detail. For one host colony, we compared the behavior of *Eciton burchellii foreli* (Formicidae: Dorylinae) workers towards specialists and generalists and analyzed the cuticular hydrocarbons (CHCs) of the ants and their guests. CHCs play a central role in ant communication [19, 38–40]. Mimicking those cues is a widespread strategy among myrmecophiles [18, 19, 28], which has been shown to facilitate their social integration [41]. Finally, we discuss chemical mimicry as an integration strategy and discuss a trade-off between level of specialization and host specificity in army ant-associated parasites.

## Methods

### Specimen collection and depository

The study took place in the tropical rainforest at La Selva Biological Station, Costa Rica (N10°25.847’ W84°00.404′, altitude 67 m asl) in an area of approximately 11 km^2^ from February to April 2013, March to April 2014, and October 2015. We collected myrmecophiles from colonies of all six local *Eciton* species during nocturnal army ant emigrations to new bivouac sites using aspirators and forceps. Compared to previous studies [20–23], we analyzed additional colony emigration samples increasing our sample size to 13 colonies of *E. burchellii foreli* Mayr, 1886 [42], 13 colonies of *E. hamatum* Fabricius, 1781 [43], eight colonies of *E. vagans angustatum* Roger, 1863 [44], 11 colonies of *E. dulcium crassinode* Borgmeier, 1955 [45], 11 colonies of *E. mexicanum* s. str. Roger, 1863 [44], and two colonies of *E. lucanoides conquistador* Weber, 1949 [46]. In addition to collections from colony emigrations, we haphazardly collected myrmecophiles from army ant raids and army ant refuse deposits. Collection details are given in Additional file 1: Table S1. This extensive community sampling together with DNA barcoding and morphological identifications of myrmecophiles allowed us to assess host preferences in great detail (see [20, 21]).

Samples from a single colony of the swarm-raiding army ant *Eciton burchellii foreli* (Additional file 1: Table S1) were used to study the chemical and behavioral integration of beetle specialists and beetle generalists. Wasmannian mimics (specialists) were identified to species-level using the identification keys of Kistner & Jacobson [32] and Reichensperger [47, 48]. *Tetradonia* beetles (generalists) were identified using the species key of von Beeren et al. [20]. Ants were identified using the identification keys of Watkins [49, 50] and Longino [51]. Army ant workers are vouchered in CvB’s personal collection. Wasmannian mimics are deposited at the Kyushu University Museum, Fukuoka, Japan (KUM) as well as in CvB’s private collection (see Additional file 2: Table S2). Additionally, 83 voucher images of 21 Wasmannian mimic specimens are deposited in the Barcode of Life database (see Additional file 2: Table S2). Taxonomy, genetics and host-specificity of *Tetradonia* beetles have been published previously [20].

### Genetic analysis

Using specimens from our community sampling approach, we determined species boundaries in Wasmannian mimics by analyzing the classical animal barcoding gene *cytochrome oxidase I* (*COI*) for 123 specimens collected from 25 colonies, including some specimens collected from army ant raids (Additional file 2: Table S2). DNA extractions and polymerase chain reactions (PCRs) were set up as described previously [20, 21]. All specimens were preserved during this process and are kept as vouchers (for depository information see Additional file 2: Table S2). The primers LCO1490 and HCO2198 [52] worked reliably, resulting in a 658 base pair (bp) fragment. Purification of PCR products and sequencing was outsourced to Macrogen USA (New York City, USA). All PCR products were sequenced in both directions. All sequences were 658 bp full length reads.

We supplemented the analysis of mitochondrial DNA (mtDNA) with nuclear DNA (nDNA), because mtDNA alone can be problematic for a genetic assessment of species boundaries (e.g., [53]). Hence, we additionally analyzed portions of the nuclear genes *wingless* (*Wg*; sequence lengths ranging from 389 bp to 480 bp) and *CAD* (sequence lengths ranging from 483 bp to 653 bp) for a subset of specimens (*Wg*: 30 specimens; *CAD*: 50 specimens; see Additional file 2: Table S2). PCRs were setup as described previously [20–23]. Primer pairs for *Wg* and *CAD* are given in Additional file 3: Figure S1. For the collection of nuclear sequence data we chose 2–18 specimens of each *COI* cluster.

DNA extraction and PCR settings as well as sequencing results were tracked for individual samples using the software Geneious® R10 (version 10.2.2) with the plugin ‘biocode’ (version 3.0.1) [54, 55]. Geneious® was also used to trim sequences and to perform Neighbor-Joining (NJ) clustering analysis with bootstrap support (1000 replicates) based on Tamura-Nei distances. NJ trees were used to screen for distinct genetic clusters within the dataset. We rooted NJ trees by using the aleocharine beetle *Ecitoglossa* sp. (GenBank accession number: MG191453), collected in a *Neivamyrmex pilosus* emigration at La Selva, as a phylogenetic outgroup (Additional file 2: Table S2). *Ecitomorpha* cf. *nevermanni* (KX586175), previously described as *Ecitomorpha arachnoides* [20], served as outgroup for the NJ tree of *Tetradonia* beetles. For *COI* data, we calculated p-distances in pairwise comparisons with pairwise deletion of missing sites using MEGA 6 [56]. All sequences are deposited in GenBank, with accession numbers listed in Additional file 2: Table S2.

### Behavioral assays

Parasites collected from one *E. burchellii foreli* colony were tested in behavioral assays (Additional file 1: Table S1). We studied *E. burchellii foreli* behavior towards specialists (46 specimens of Wasmannian mimics), generalists (14 specimens of *Tetradonia*) and 10 heterospecific *E. hamatum* intermediate workers in a laboratory nest setup (plastic box size: 25 cm*15 cm*5 cm) with a plaster of Paris floor. The term intermediate refers to middle-sized *Eciton* workers, which were previously also denoted as medias [57] or medium workers [31]. Furrows were scratched into the hardening plaster with a fork to provide hiding spots for parasites. Nest boxes contained approximately 100 *E. burchellii foreli* workers, including workers of all sizes. A subset of these workers was later used for chemical extractions.

Test animals were introduced one at a time. Each specimen was given a settling time of 30 s, after which we observed its interactions with *E. burchellii foreli* workers for one minute. We counted the number of contacts of test specimens with *E. burchellii foreli* workers. A contact was defined as physical contact between any body part of the focal test animal and an *E. burchellii foreli* worker. This included short-lasting interactions such as antennal touch as well as long-lasting contacts such as reciprocal grooming (see [58] for possible ant-myrmecophile interactions). Lasting contacts with the same individual ant were only counted once. In addition, we counted the number of aggressive ant behaviors (chasing, snapping, stinging attempt) towards test specimens. Aggressive ant behaviors were defined previously [41, 59].

**Fig. 1:**
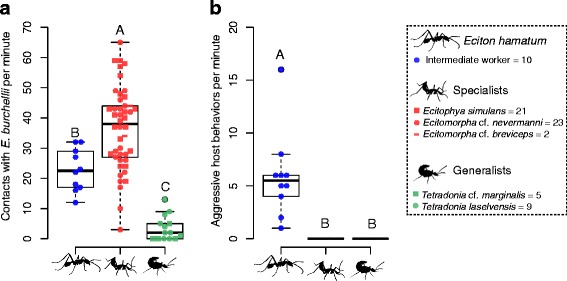
Behavioral assays. **a** The number of contacts between *Eciton burchellii foreli* workers with test specimens and **b** the number of aggressive host behaviors towards test specimens. No aggressive host behavior was detected against Wasmannian mimics (specialists) and *Tetradonia* beetles (generalists), and individual data points are therefore not plotted. Different letters depict significant differences (*p* < 0.05) as assessed by a Dunn’s test

Behavioral assays were analyzed in R 3.3.1 [60]. The number of contacts and the number of aggressive behaviors of *E. burchellii foreli* workers towards *E. hamatum* intermediates, Wasmannian mimics (specialists) and *Tetradonia* beetles (generalists) were compared using Kruskal-Wallis tests [61]. We did not find differences in contact frequency with host ants between different species of Wasmannian mimics (Kruskal-Wallis test, χ^2^ = 3.16, df = 2, *P* = 0.205; Fig. 1a) or between different *Tetradonia* species (Kruskal-Wallis test, χ^2^ = 2.15, df = 1, *P* = 0.131; Fig. 1a). Specimens of the different species were therefore pooled into the categories specialists and generalists, respectively. The same applies for the analysis of ant aggression (Fig. 1b). As post-hoc analyses we used Dunn’s multiple comparisons rank sum test [62] with false discovery rate correction to account for type I error-accumulation [63] as implemented in the R package PMCMR [64].

### Chemical analysis

The same individuals studied in behavioral assays were chemically analyzed. Chemical extractions of specimens took place within a period of 1–15 min after the behavioral test. We extracted the cuticular hydrocarbons (CHCs) of ant specimens (10 minor, 9 intermediate, 10 major workers, and 10 larvae of *E. burchellii foreli*, and 11 intermediate workers of *E. hamatum*), 45 Wasmannian mimics (specialists), and 14 *Tetradonia* beetles (generalists). *Eciton hamatum*, an heterospecific, congeneric army ant species, was used as a chemical outgroup. Each specimen was submersed in 200 μl n-hexane (98% purity for gas-chromatography) at room temperature for 10 min. Subsequently, the animals were removed from the solvent and preserved in alcohol for genetic and morphological analyses. The hexane was then evaporated at room temperature in a fume-hood.

The CHC residuals were re-dissolved in 40 μl n-hexane containing hexadecane as internal standard. The concentrations of our internal standard were adjusted according to body size. We used 5 ng hexadecane / 1 μl hexane for Wasmannian mimics, *Tetradonia* beetles, and all ants except for *E. burchellii foreli* majors, for which we used 15 ng/μl. Then, 20 μl of the samples were transferred into conical glass inlets. We analyzed the samples with a QP 2010ultra GC-MS (Shimadzu, Japan). The gas chromatograph was equipped with a ZB-5MS fused silica capillary column (30 m × 0.25 mm ID, df = 0.25 μm) from Phenomenex (USA). An AOC-20i autosampler-system from Shimadzu was used to inject 1 μl sample aliquot into a programmed temperature vaporizing split/splitless-injector (Optic MultiMode Inlet 4, GL Sciences, Netherlands), which operated in splitless-mode. Injection temperature was programmed from initially 50 °C (5 s hold) to 300 °C with a heating-rate of 50 °C/s and a subsequent hold for 59 min. Hydrogen was used as carrier-gas with a constant flow rate of 1.3 ml/min. The temperature of the GC oven was raised from an initial 60 °C for 1 min, to 320 °C with a heating-rate of 5.5 °C/min and then an isothermal hold at 320 °C for 10 min. Electron ionization mass spectra were recorded at 70 eV with a scan rate of 2 scans per sec from *m/z* 40 to 650. The ion source of the mass spectrometer and the transfer line were kept at 230 °C and 300 °C, respectively.

Pooled extracts of 10 ant workers were used for compound identification. The CHCs were identified based on their *m/z* fragmentation patterns and gas chromatographic retention indices (RI), which were calculated using an alkane standard mixture (C7-C40 dissolved in hexane; Sigma-Aldrich, Germany) using the method of van den Dool & Kratz [65]. The structural identities of methyl-branched alkanes were assigned according to established procedures [66, 67]. The double bond positions in alkenes and alkadienes were identified using iodine-catalyzed dimethyldisulfide derivatization [68]. The configurations of double bonds were not determined.

CHCs of ants and myrmecophiles were analyzed as compositional data (i.e. percentages of CHCs per specimen). For each specimen, CHCs below a threshold of 5% of the total CHC amount were not considered to account for a potential bias caused by assaying animals of vastly different body sizes. The CHC compositional data were analyzed using discriminant analysis of principal components (DAPC). DAPC is a powerful method to discriminate a priori assigned groups in a multivariate ordination of chemical compositional data [69]. It transforms the original compositional data by PCA prior to the discriminant analysis (DA) and therefore values become uncorrelated [70]. We retained three PC-axes based on their Eigenvalues and the explained variance. We further used PERMANOVA [71] based on 10,000 permutations with Bray-Curtis similarities [72] to test for compositional differences of CHC profiles among groups and PERMDISP [69, 71] to test the compositional stability of CHC profiles among ants, beetle specialists, and beetle generalists. Permutational analysis of variance (PERMANOVA) and permutational analysis of multivariate dispersions (PERMDISP) were run with the R packages ˈadegenetˈ [73] and ˈveganˈ [74], respectively. PERMANOVA pair-wise tests and PERMDISP pair-wise tests were run with the software Primer 7 (Primer-E Ltd., Ivybridge, U.K., vers. 7.0.12) with the add-on PERMANOVA+ 1 [75]. We did detect differences in CHC composition among species of Wasmannian mimics (PERMANOVA, Pseudo-F = 9.545, *P* < 0.001; PERMDISP, F = 1.357, *P* = 0.829) and between *Tetradonia* species (PERMANOVA, Pseudo-F = 3.941, *P* < 0.012; PERMDISP, F = 0.194, *P* = 0.389). However, we still pooled the different species into the respective categories being aware that minor differences existed among species within groups, because the aim of the present study was to compare two distinct groups of ant parasites: beetle specialists and beetle generalists.

The amounts of CHCs [in ng] were calculated based on the internal standard hexadecane as described in [76]. To consider differences in body size among specimens we either standardized using the body mass [in mg dry weight; see Additional file 3: Figure S2 for all dry weight measurements] or the estimated surface area with the obtained dry weight data [in mg^2/3^] (see [77]). The latter analysis accounts for the faster increase of volume compared to surface area (in this study the dry weight) when a size of an object increases (square-cube law; [78]). The frequent exchange of CHCs among ant nestmates creates a uniform colony odor, the so-called *gestalt* odor [19, 79]. Owing to the *gestalt* odor, we expected to find a positive relationship between CHC amount and body size in ants. Indeed, we found a linear relationship between body mass and CHC amount of *E. burchellii foreli* workers including specimens from different castes (*N* = 28, F-value: 113, *P* < 0.001; Additional file 3: Figure S3). This indicated that the dry weight is a good predictor for an animal’s surface area and thus can be used to assess the CHC concentration, assuming a relatively constant shape of the study object. To obtain the body mass, we dried the specimens until weight constancy for at least 48 h at 45 °C and determined their dry weight using a microbalance (Mettler Toledo, XS3DU, USA). The CHCs per dry weight [ng/mg] or per estimated surface area [ng/ body mass^2/3^] were analyzed using Kruskal-Wallis tests and affiliated Dunn’s tests as described above. No differences were found among species of Wasmannian mimics (ng/mg: Kruskal-Wallis test, χ^2^ = 4.367, df = 2, *P* = 0.113; ng/mg^2/3^: Kruskal-Wallis test, χ^2^ = 3.416, df = 2, *P* = 0.181) or between *Tetradonia* species (ng/mg: Kruskal-Wallis test, χ^2^ = 0.751, df = 1, *P* = 0.386; ng/mg^2/3^: Kruskal-Wallis test, χ^2^ = 0.111, df = 1, *P* = 0.739). Specimens of the different species were pooled into the categories specialists and generalists, respectively.

## Results

### Genetic assessment of species boundaries

**Fig. 2:**
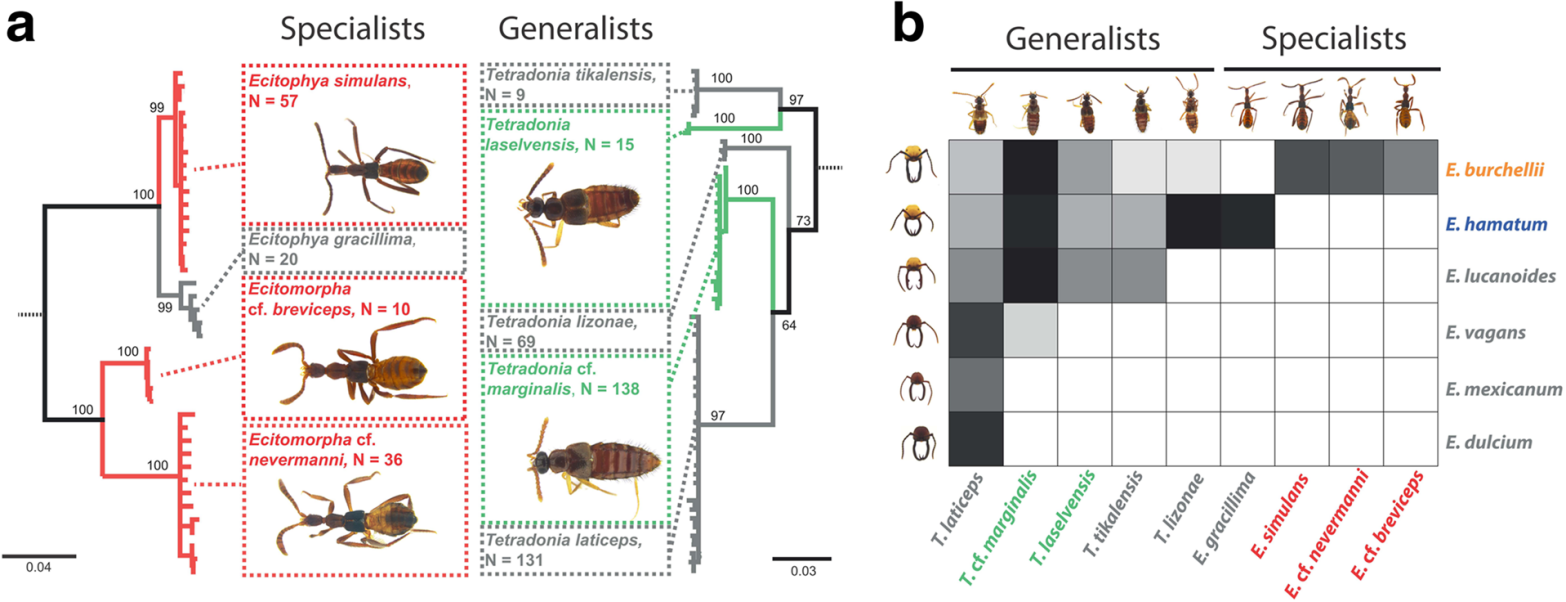
Genetic assessment of parasite species boundaries and parasite host preferences. Coloration depicts species that were also studied in behavioral and chemical analysis (red = specialists; green = generalists; orange = *E. burchellii foreli*; blue = *E. hamatum*; see also other figures). Grey boxes depict species that were not considered further. **a** Neighbor-joining trees based on Tamura-Nei distances (scale bars) of the mitochondrial gene fragment *COI* detected four genetic clusters for *Ecitophya* and *Ecitomorpha* (specialists) and five genetic clusters for *Tetradonia* (generalists). We excluded the genetic outgroups from the NJ trees for better visibility (indicated by dashed lines; outgroups: *Ecitoglossa* sp. for specialists and *Ecitomorpha* cf. *nevermanni* for generalists). Numbers of analyzed specimens is given in boxes. Bootstrap support values are shown at major branches (1000 replicates). **b** Host specificity of *Tetradonia* beetles (generalists) and Wasmannian mimics (specialists). Differential shading corresponds to the proportion of colonies of a given host species in which a given parasite species was collected. White boxes depict missing associations between parasites and army ants. Black boxes show myrmecophiles that were collected in all colonies of a given host species. Photographs depict frontal head views of *Eciton* soldier workers and dorsal views of beetles for the different species. Data on *Tetradonia* species boundaries and host specificity have been published previously [20]. Specimen images are not to scale in both graphs

Genetic assessment of species boundaries revealed the presence of four candidate species of Wasmannian mimics (specialists) and five candidate species of *Tetradonia* beetles (generalists) in the *Eciton*-myrmecophile community at La Selva (Fig. 2a; for detailed results about *Tetradonia* beetles see [20]). The NJ-tree clustering analysis revealed four distinct genetic clusters in Wasmannian mimics (Fig. 2a). The four *COI* clusters were also recovered with the nuclear loci *Wg* and *CAD* (Additional file 3: Figure S1). There was no haplotype overlap between *COI* clusters (within clusters: *E.* cf. *breviceps*: 4 haplotypes, *N* = 10 specimens sequenced; *E.* cf. *nevermanni*: 12 haplotypes, *N* = 36 specimens sequenced; *E. gracillima*: 5 haplotypes, *N* = 20 specimens sequenced; *E. simulans*: 16 haplotypes, *N* = 57 specimens sequenced). Similarly, there was no sequence overlap between genetic clusters in *CAD* or in *Wg* (number of *CAD*/*Wg* sequences: *E.* cf. *breviceps* = 2/3; *E.* cf. *nevermanni* = 14/15; *E. gracillima* = 18/2; *E. simulans* = 16/9). The distribution of intraspecific- and interspecific genetic p-distances in pairwise comparisons of *COI* sequences revealed a gap between maximum intraspecific- and minimum interspecific genetic distances of mitochondrial sequences (Additional file 3: Figure S4). Such ‘barcoding gaps’ provide additional support for the presence of distinct species (but see [53]).

### Taxonomy and host specificity

Guided by the molecular work, we were able to reliably distinguish the four candidate species of beetle specialists on the basis of several anatomical characters. We identified them as the following species: *Ecitophya simulans* Wasmann 1889, *Ecitophya gracillima* Mann 1925, *Ecitomorpha* cf. *nevermanni* Reichensperger 1935 and *Ecitomorpha* cf. *breviceps* Reichensperger 1933.

*Ecitophya simulans* and *E. gracillima* are similar to each other externally, but they are clearly distinguished in the head capsule and antennal shapes. In *E. simulans* the head length is less than twice as long as wide (head length/ head width = 1.91–1.93), and the antennal segment II is about half as long as the segment X, while in *E. gracillima* the head length is more than twice as long as wide (head length/ head width = 2.26–2.30), and the segment II is less than 1/3 of the segment X. The two species can also be distinguished by their color. The abdominal tergite VI of *Ecitophya simulans* is almost uniformly reddish brown, but that of *E. gracillima* is clearly dark with brownish tinge around middle area. The two *Ecitomorpha* species are easily distinguishable by the shape of the pronotal incision, the absolute and relative length of antennal segments, and the shape of the head (for details see Additional file 3: Figure S5). Note that *Ecitomorpha* formally contains a single species (i.e. *E. arachnoides* Wasmann 1889) [32]. In the latest revision of Ecitocharini, Kistner & Jacobson synonymized the species *E. arachnoides*, *E. melanotica* Mann 1926, *E. breviceps* Reichensperger 1933, and *E. nevermanni* Reichensperger 1935 [32]. However, our genetic and morphological analyses revealed the presence of two distinct *Ecitomorpha* species, which matched well to the species descriptions of *E. breviceps* and *E. nevermanni* (Additional file 3: Figure S5). We therefore decided to refer to the species studied here as *Ecitomorpha* cf. *nevermanni* and *Ecitomorpha* cf. *breviceps* (cf. = confer).

Each species of Wasmannian mimic was specifically associated with a single host (Fig. 2b). *Ecitophya gracillima* was exclusively associated with *E. hamatum* (Fig. 2b)*.* It was found in 12 out of 13 *E. hamatum* colonies from which myrmecophiles were collected systematically. *Ecitophya simulans*, *Ecitomorpha* cf. *breviceps*, and *Ecitomorpha* cf. *nevermanni* were exclusively collected from *E. burchellii foreli* colonies and were present in 11, 7, and 9 out of 13 systematically sampled colonies, respectively. Wasmannian mimics were not found with any of the other *Eciton* species occurring at La Selva (*E. dulcium*, *N* = 11 sampled colonies; *E. lucanoides*, *N* = 2; *E. mexicanum*, *N* = 11; *E. vagans*, *N* = 8). Note that another Wasmannian mimic, the beetle *Ecitophya rettenmeyeri* Kistner & Jacobson 1990, was previously collected at La Selva from *E. lucanoides* but was not detected in the current samples. Figure 2b also reviews species boundaries and host specificity of *Tetradonia* beetles. These data have been published previously [20].

### Behavioral assays

We observed that Wasmannian mimics actively sought contact with host ants and often engaged in reciprocal grooming behavior with workers (see Additional file 4). In contrast, *Tetradonia* beetles avoided host contact and mostly hid in gypsum cavities. Congeneric *E. hamatum* workers also avoided contact but were regularly seized by *E. burchellii foreli* workers. Accordingly, we found differences among groups in host contact frequency (Kruskal-Wallis test, χ^2^ = 37.84, df = 2, *P* < 0.001; Fig. 1a). Wasmannian mimics (specialists) and *E. hamatum* workers interacted more often with *E. burchellii foreli* workers in behavioral assays than *Tetradonia* beetles (generalists) (mean number of contacts per minute ± SD; *E. hamatum*: 23 ± 7, *N* = 10; specialists: 37 ± 14, *N* = 46; generalists: 3.5 ± 4, *N* = 14; Fig. 1a).


**Additional file 4:** Interactions between Wasmannian mimics and army ant host workers. Video credit: Philipp O. Hönle. (MP4 34 mb)


Host aggression against test specimens also differed among groups (Kruskal-Wallis test, χ^2^ = 68.47, df = 2, *P* < 0.001; Fig. 1b). Congeneric *E. hamatum* workers (*N* = 10) were aggressed by *E. burchellii foreli* workers (mean ± SD: 6 ± 4 aggressive events per minute), while we detected no aggression towards Wasmannian mimics and *Tetradonia* beetles (Fig. 1b). During field and additional laboratory observations, however, we did occasionally observe aggression towards *Tetradonia* beetles, but not towards Wasmannian mimics.

### Chemical profiles of hosts and myrmecophiles

Workers of *Eciton burchellii foreli* and *Eciton hamatum* army ants possessed simple cuticular chemical profiles dominated by three main compounds (Table 1): heneicosane (C21), tricosane (C23) and 9-tricosene (C23–9-ene). Similar CHCs were found in Wasmannian mimics (specialists) and *Tetradonia* beetles (generalists) (Table 1). No additional, myrmecophile-specific CHCs were detected (Table 1).

**Table 1:** Relative abundances of CHC compounds

CHC identification	RI	Generalists		Specialists			*Eciton burchellii foreli*				*Eciton hamatum*
		*Tetradonia laselvensis*	*Tetradonia* cf. *marginalis*	*Ecitomorpha* cf. *breviceps*	*Ecitomorpha* cf. *nevermanni*	*Ecitophya simulans*	larva	minor	inter-mediate	major	intermediate
C21–6,9-diene	2069	–	–	–	–	–	–	–	–	–	0.02
C21–9-ene	2075	–	–	0.95	0.82	0.86	–	1.27	1.39	0.92	0.99
C21–7-ene	2080	–	–	–	0.11	–	–	0.04	–	0.04	0.05
C21	2100	21.73	17.48	22.33	20.72	16.43	17.43	19.29	16.34	8.04	15.24
11-Me-C21	2138	–	–	–	–	–	–	–	–	0.03	0.13
9-Me-C21	2142	–	–	–	–	–	–	–	–	–	0.01
C22–9-ene	2174	–	–	–	0.08	0.60	–	0.10	0.11	0.18	0.15
C22–7-ene	2181	–	–	–	–	0.18	–	–	–	–	0.01
C22	2200	2.32	1.07	1.37	1.31	1.01	0.29	1.26	0.71	0.19	0.32
C23–6,9-diene	2271	–	–	0.46	0.77	0.93	0.56	–	0.49	0.77	0.19
C23–9-ene	2286	24.06	32.83	38.09	46.60	54.74	57.33	44.82	58.23	80.33	75.19
C23–7-ene	2289	0.96	5.07	2.20	2.26	2.40	1.30	2.02	1.04	1.00	0.15
C23:1	2295	0.28	0.35	0.69	0.37	0.38	0.00	0.23	0.22	0.21	0.02
C23	2300	38.81	35.85	26.25	21.98	16.73	19.21	22.82	15.79	6.04	6.42
Me-C23	2324	–	–	–	–	–	–	–	–	0.07	–
11-Me-C23	2335	0.55	1.03	0.95	0.52	0.61	0.16	0.89	0.49	0.22	0.05
9-Me-C23	2341	0.20	0.21	–	0.04	0.10	–	0.33	0.23	0.05	0.01
C24	2400	0.49	–	–	0.09	0.12	–	0.37	0.21	0.04	0.01
C25–6,9-diene	2469	–	–	–	–	–	–	–	–	0.01	0.00
C25–9-ene	2475	0.20	0.99	1.31	0.87	1.54	0.18	0.94	0.88	0.73	0.14
C25–7-ene	2484	–	–	0.18	0.31	0.32	–	0.15	0.22	0.18	0.01
C25	2500	10.38	5.11	5.21	3.12	2.79	3.55	4.62	2.72	0.63	0.32
11-Me-C25	2535	–	–	–	–	–	–	–	–	0.03	–
C26	2600	–	–	–	–	–	–	–	–	0.01	–
C27–9-ene	2675	–	–	–	–	–	–	–	0.02	0.01	–
C27–7-ene	2684	–	–	–	–	–	–	–	–	0.01	–
C27	2700	–	–	–	0.03	0.21	–	0.78	0.57	0.12	0.21
11-Me-C27	2772	–	–	–	–	–	–	–	–	tr	–
C29–6,9-diene	2870	–	–	–	–	–	–	–	–	tr	–
C29–9-ene	2875	–	–	–	–	–	–	–	–	tr	–
C29–7-ene	2886	–	–	–	–	–	–	–	–	tr	–
C29	2900	–	–	–	–	0.04	–	–	0.20	0.06	0.17
C30:1	2985	–	–	–	–	–	–	0.07	–	0.02	–
C31:1	3050	–	–	–	–	–	–	–	–	0.01	0.11
C31:1	3076	–	–	–	–	–	–	–	–	–	0.02
C31	3100	–	–	–	–	–	–	–	0.11	0.03	0.06
Me-C31	3145	–	–	–	–	–	–	–	–	0.01	–

Relative abundances of individual components to the specimen’s total peak area are given for different *Eciton burchellii foreli* castes, different parasite species, and *Eciton hamatum* intermediate workers. Values represent means of each category (for sample sizes for each category see Results). Abbreviations: *RI* retention index, *tr* traces detected, − not detected

**Fig. 3:**
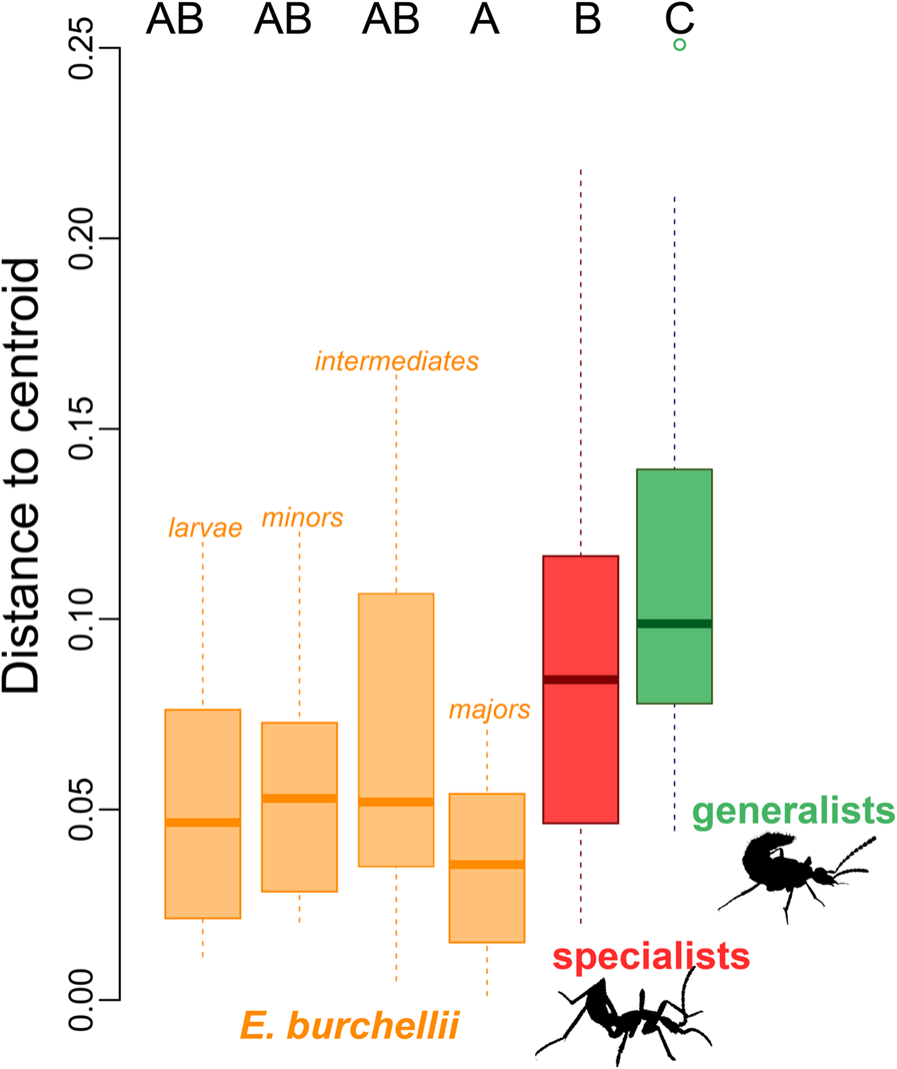
Dispersion of CHC profiles within categories. Distance to centroid in CHC compositional data (based on Bray-Curtis similarities) for the categories larvae (*N* = 8), minor workers (*N* = 10), intermediate workers (*N* = 9), major workers (N = 10), Wasmannian mimics (specialists; *N* = 44) and *Tetradonia* beetles (generalists; *N* = 14). Capital letters depict significant differences in permutational pairwise comparisons (PERMDISP pairwise test; *P* < 0.05)

CHC composition differed among the following groups: 'specialist', 'generalist', '*E. hamatum*', '*E. burchellii* major', '*E. burchellii* intermediate', '*E. burchellii* minor', and '*E. burchellii* larva' (PERMANOVA, Pseudo-F = 43.13, *P* < 0.001). In PERMANOVA pairwise comparisons all groups differed from each other (PERMANOVA, *P* ≤ 0.031) except for Wasmannian mimics (specialists) and *E. burchellii* larvae (PERMANOVA, *P* = 0.141). We also detected differences in the within-group variance (PERMDISP, F = 4.449, *P* = 0.003) with CHC compositional data being more dispersed in *Tetradonia* beetles than in ants and Wasmannian mimics (Fig. 3).

**Fig. 4:**
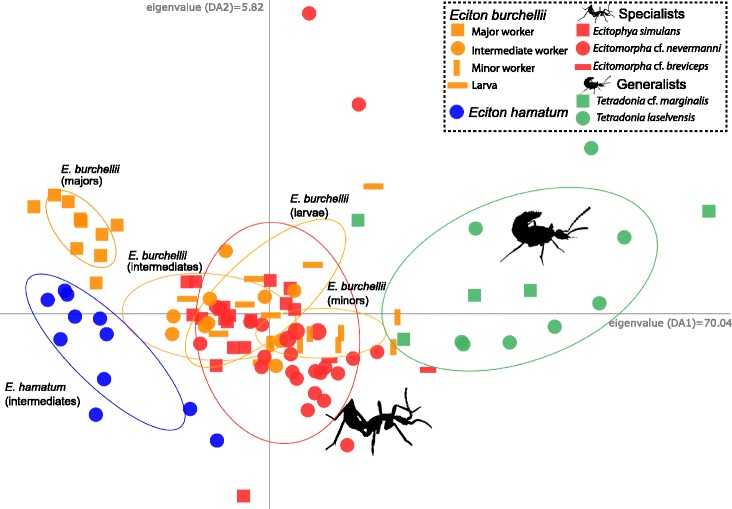
CHC profile similarity between ants and parasites. Discriminant analysis of principal components of CHC profiles for *E. burchellii foreli* (larvae *N* = 8, minor workers *N* = 10, intermediate workers *N* = 9, major workers *N* = 10), *E. hamatum* intermediate workers (*N* = 11), beetle specialists (*N* = 45), and beetle generalists (*N* = 14). Eigenvalues are shown at the tip of axes. Ellipses depict 95% confidence intervals of different categories

The DAPC ordination provided strong evidence that chemical profiles of host ants were qualitatively very similar to the profiles of specialists (Fig. 4). Apart from one minor worker, a DAPC reassignment of a priori defined groups could not distinguish CHC profiles of larvae, minor workers and intermediate workers of *E. burchellii foreli* from CHC profiles of 'specialists' (Additional file 3: Table S3). In contrast, *E. burchellii foreli* major workers and *Tetradonia* beetles (generalists) had distinct chemical profiles (Fig. 4) and were robustly assigned to their respective group by the DAPC (Additional file 3: Table S3).

**Fig. 5:**
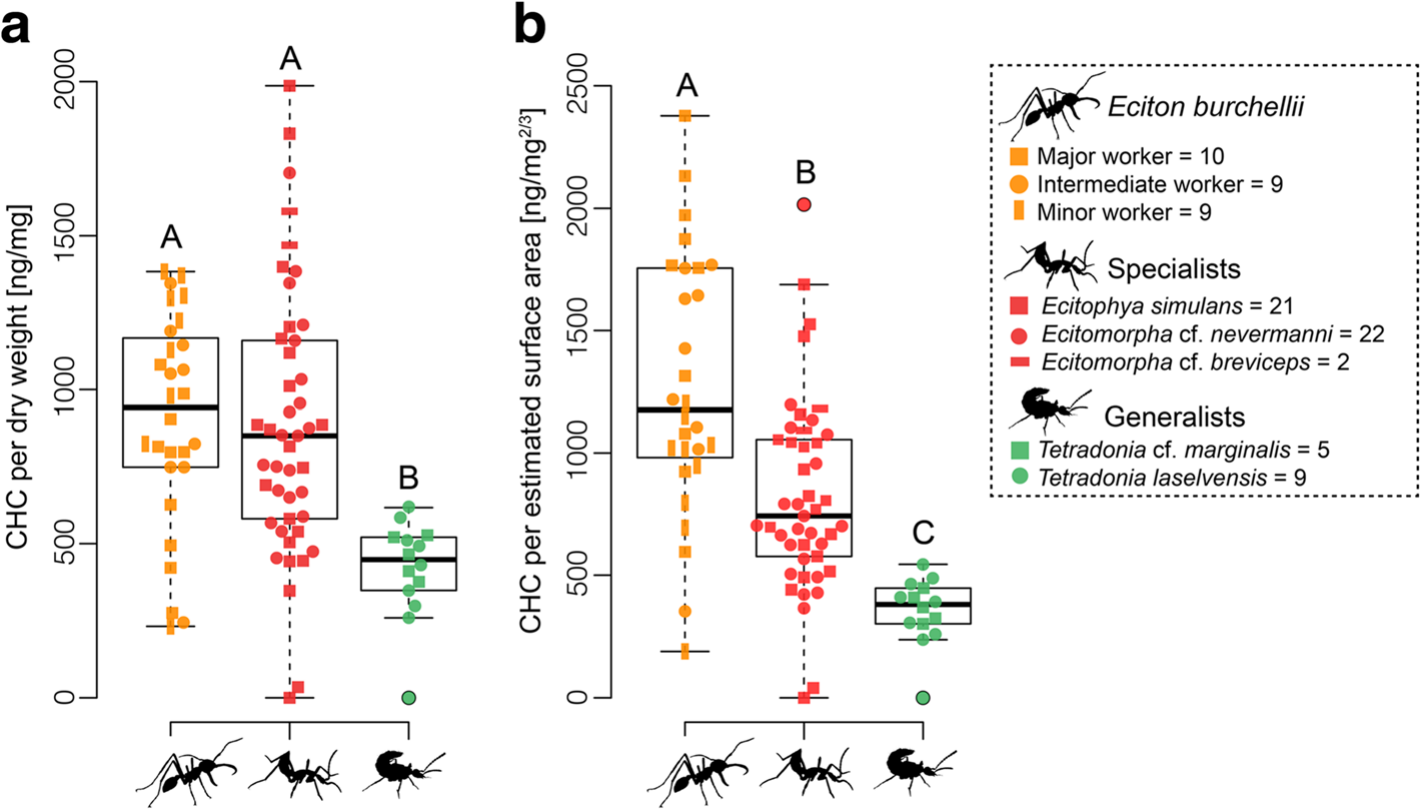
Army ant and parasite CHC concentrations. CHC amount per dry weight (**a**) and CHC amount as a function of estimated surface area (**b**) plotted for *Eciton burchellii foreli* workers, beetle specialists, and beetle generalists. Sample sizes are given in the figure legend. Different letters depict significant differences (*p* < 0.05) as assessed by a Dunn’s test

The CHC concentration also differed among the groups (for ng/mg: Kruskal-Wallis test, χ^2^ = 19.66, df = 2, *P* < 0.001, Fig. 5a; for ng/mg^2/3^: Kruskal-Wallis test, χ^2^ = 35.62, df = 2, *P* < 0.001, Fig. 5b). Depending on the method used for body size correction, the CHC concentration of ant workers and beetle specialists either did not differ when standardizing the CHC amount for dry weight (Dunn’s post hoc test, *P* = 0.620; Fig. 5a) or it differed for the estimated surface area in that beetle specialists had lower CHC concentrations than ants (Dunn’s post hoc test, *P* = 0.002; Fig. 5b). Beetle generalists showed lower CHC concentrations than workers and beetle specialists, irrespective of the method used for body size correction (Dunn’s post hoc test for both analyses, *P* < 0.001; Fig. 5a,b).

## Discussion

Community sampling of army ant myrmecophiles, combined with an integrated study of beetle taxonomy, unveiled the presence of four *Eciton*-associated species of Wasmannian mimics at La Selva, Costa Rica. Each of these specialized mimics was specifically associated with a single host species. We compared the chemical and behavioral integration of these specialists with a group of beetle generalists, i.e. *Tetradonia* beetles. We found that specialists showed a higher level of social integration and a closer match to host CHC profiles.

### Assessing species boundaries

Communities of army ant myrmecophiles are species-rich [5, 31]. To efficiently determine myrmecophile species boundaries, we applied a molecular pre-screening mechanism using classical DNA barcoding of the mitochondrial locus *COI* combined with the analysis of two nuclear loci [20–23]. Distinct *COI* clusters that were also recovered with nuclear loci were considered to represent candidate species (see also [53]), which were subsequently inspected morphologically. This approach already helped us distinguish species in other army ant associates [22, 23], including taxonomically difficult groups such as *Vatesus* (Staphylinidae: Tachyporinae) and *Tetradonia* beetles [20, 21].

In the present study, we determined species boundaries in *Eciton*-associated *Ecitophya* and *Ecitomorpha* beetles. Genetic and morphological inspection agreed on the presence of four distinct species in the La Selva community. Two of these species were members of the genus *Ecitomorpha,* which formally contains only a single species (see latest revision [32]). It seems likely that these two species had been previously described as *Ecitomorpha nevermanni* [47] and *Ecitomorpha breviceps* [80] and were later mistakenly synonymized by Kistner & Jacobson [32]. However, a validation of these tentative species identifications, including careful morphological inspection of type material, is necessary.

A recent genetic study [33] likewise indicated cryptic diversity in *Ecitophya* and *Ecitomorpha*. The study revealed strong intrageneric genetic divergence in beetles associated with two distinct host subspecies, *Eciton burchellii parvispinum* and *Eciton burchellii foreli*, which were studied in West and Central Panama, respectively. However, given the lack of morphological data in that study, it remains to be determined whether the detected genetic differences are in fact continuous and derive from limited gene flow due to the geographic distance separating the two study areas, or whether the two populations indeed represent reproductively isolated populations and, therefore, different species (see also discussion in [22]). Either way, these results and the data presented here certainly call for a careful taxonomic revision of this fascinating group of army ant-associated rove beetles, which would ideally include samples covering the beetles’ distributional range to more reliably assess species boundaries (see [33] and discussion in [22]).

### Host specificity of Wasmannian mimics

The level of host specificity plays a fundamental role in symbiotic interactions [10, 11, 81, 82]. However, its assessment can be challenging [10, 11]. It requires sampling of a community of possible host species and a correct delimitation of species boundaries [10, 13, 21]. Previous host records of Wasmannian mimics, however, were either based on haphazard collections from different populations, or considered only a fraction of potential host species in a given community [27, 32, 33, 83]. Even though they suggested an association with a single host species, this conclusion remained questionable [27, 32, 83]. Combining our extensive collection of myrmecophiles from large numbers of *Eciton* colonies within a single community with the genetic and morphological assessment of species boundaries allowed us to reliably determine host specificities, confirming a single host association in four species of Wasmannian mimics.

### Resemblance to host CHC profiles – A comparison between specialists and generalists

Almost 100 years after Wasmann hypothesized that ant-mimicking beetles resemble ants chemically [37] we can confirm his supposition (see also [84]). The close resemblance to host CHC profiles in Wasmannian mimics most likely constitutes a case of chemical mimicry (sensu [85]), which implies two things. First, Wasmannian mimics are detected by host ants as interesting entities, which is, for example, not the case in chemical crypsis (background matching) and chemical masquerade (matching of an uninteresting entity) (reviewed in [85]). Frequent interactions between hosts and Wasmannian mimics, including reciprocal antennation and grooming, demonstrated that ants perceived the guests as interesting entities. The first requirement to denote this association as mimetic is thus fulfilled, but we can only speculate about the second one: mimics need to benefit from a deception of the operator (here the host ants) [85, 86]. Since CHC profiles in ants mediate nestmate recognition and the detection of alien species [38, 40, 87–90], mimicking those cues likely hampers the ants’ recognition of mimics as heterospecific intruders. We consider it likely that ants mistake Wasmannian mimics as conspecific nestmates, a type of deception that has previously been suggested for various other ant-myrmecophile interactions [9, 18, 19]. However, to denote this resemblance as chemical mimicry requires evidence of host deception, for example, via manipulative experiments of the chemical cues as demonstrated in an army ant-associated silverfish [41]. Future studies should thus consider altering the CHC profiles of Wasmannian mimics experimentally to see whether the host behavior towards parasites is indeed influenced by the level of CHC similarity (e.g., [41, 58]).

The same line of argument applies to beetle generalists. Compared to Wasmannian mimics, these generalists resembled the host’s CHC profile less well and showed a higher profile variation and lower CHC concentrations, which might partly explain their lower level of social integration. Imperfect CHC mimicry in myrmecophile-ant systems is common, but the ultimate and proximate mechanisms behind a weak chemical host resemblance mostly remain speculative. Carrying only small quantities of CHCs compared to host ants has often been suggested to hamper recognition due to an inability of host ants to detect these cues (chemical insignificance sensu [19]; chemical hiding sensu [85]) (e.g., woodlice/mites/phorid flies: [91]; syrphid flies: [92]; silverfish: [93]). On the contrary, the presence of only a few host CHCs in low quantities with slight compositional differences to the host has been suggested as a strategy of two parasitoid wasps to be detected by host ants [94]. In this case, the ants pick up the adult wasps and transport them out of the host nest, an essential step in the wasps’ life cycle [94].

As we did not detect any aggression of host ants towards *Tetradonia* beetles in behavioral assays, we assume that even the weak similarity in CHC profiles to host ants is beneficial to avoid an immediate recognition, which generally triggers worker hostility. In contrast to many of the unspecialized myrmecophiles of red wood ants [17], for example, the CHC profiles of *Tetradonia* beetles still resembled the CHC profiles of host ants fairly well in that they carried the exact same CHCs as host ants without possessing any idiosyncratic CHCs. The latter phenomenon is common among social insect symbionts (e.g., beetles: [95, 96]; eucharitid wasps: [94, 97]; silverfish: [41, 98]; spiders: [58, 98, 99]; syrphid flies: [92, 100]), most likely because a symbiont possessing components absent in the host cuticular profile would facilitate being recognized as an intruder. A very similar pattern to *Tetradonia*’s chemical resemblance – same CHCs as host ants in non-integrated species – was previously detected in staphylinid parasites of Southeast-Asian *Leptogenys* army ants [59, 91]. We speculate that host profile similarity in non-integrated generalists might arise as a by-product of ant predation by physical CHC transfer from host ants to predatory beetles (see also discussion in [101]). This might represent one of the first steps during the evolution of myrmecophily to lower defensive responses by ant hosts (see also [17]).

### Origin of mimetic CHCs

There are two principal mechanisms for how myrmecophiles can obtain mimetic CHCs, and these mechanisms differently affect host-symbiont co-evolutionary dynamics [9, 85]. Myrmecophiles can either acquire mimetic CHCs from host workers via physical contact (acquired chemical mimicry sensu [85]), they can synthesize mimetic cues themselves (innate chemical mimicry sensu [85]), or use a combination of both strategies (reviewed in [9, 18, 19]). Acquired chemical mimicry seems to be the most common strategy among myrmecophiles [19], and we consider it likely that this is the primary mechanism in Wasmannian mimics and *Tetradonia* beetles. Wasmannian mimics engaged in intensive and constant host contact (see also [24]), which likely transferred host CHCs to the beetles as demonstrated in another army ant-parasite system [41, 58]. *Tetradonia* beetles are predators of *Eciton* ants [24, 26] and we assume that they acquired host CHCs during the handling and feeding process. This weaker match to host profiles combined with their lower contact frequency with host ants suggests that constantly updating mimetic CHCs via host contact is necessary to establish and maintain a good match to host CHC profiles (see also [28, 41, 58]).

Besides CHC acquisition, an additional de novo synthesis of certain key compounds is likewise possible [18, 19, 96]. Experimental designs to test for de novo synthesis of mimetic cues include CHC extractions of myrmecophiles without previous host contact [102–104] and CHC extractions of myrmecophiles that were experimentally separated from host ants for an extended period of time [28, 41, 58, 93]. These experiments could be done with *Tetradonia* beetles, but carrying them out with Wasmannian mimics is difficult for two reasons. First, all adults have been collected from host colonies and immatures have not been found yet, so CHC profiles of individuals without previous host contact cannot be studied. Second, separating the beetles from host ants is impossible as they die after a few days of isolation ([24, 32], and personal observation). At this point, it remains uncertain whether innate chemical mimicry plays a role for deceiving host ants in Wasmannian mimics and *Tetradonia* beetles - a mechanism that might be particularly important during the initial contact with army ant colonies (e.g., [102, 105–107]).

### CHC concentration

Besides compositional differences in CHC profiles, CHC concentrations might also play a role in chemical communication of ants (e.g., [108]) and in chemical mimicry of myrmecophiles [17, 41, 58]. At this point, this is highly speculative, but it points the way to further research. As expected, well-integrated specialists had higher concentrations of mimetic CHCs compared to non-integrated generalists. The CHC concentration, i.e. the CHC amount per surface area or body mass, has hitherto largely been neglected as a factor for chemical integration of myrmecophiles [41, 58, 91, 95, 109]. Maybe this is because it is difficult to measure an animal’s surface area. Previous studies estimated myrmecophile surface areas using approximations of geometrical shapes [17, 41, 58, 109]. While these simplified calculations allowed to standardize an animal’s CHC amount for vastly different body sizes, such calculations can only be treated as rough estimates of an animal’s actual surface area [109]. In the present study, we used an animal’s dry weight as estimator for body size. The dry weight, and in particular the dry weight^2/3^, is generally a good predictor of an insect’s surface area [76, 77]. Ideally, future studies will assess surface areas via 3-D scans directly to calculate CHC concentrations of ants and their mimics [76, 77], but we suggest the dry weight as a reliable and feasibly measurable surrogate. Using dry weight as surface area predictor might be useful in studying both, the role of CHC concentration in ant nestmate recognition (e.g., [108]) and in the recognition of chemical mimics (e.g., [41]).

### Additional adaptations to myrmecophily

It is important to mention that integration mechanisms of myrmecophiles are multifaceted. Chemical mimicry of host CHCs is one among many possible mechanisms to cope with ants, also including morphological and behavioral adaptations [2, 3, 24, 31, 110], acoustical mimicry [29, 30, 111], and attractive or defensive chemical gland secretions [112–115]. It is the interplay between these factors that ultimately governs host-myrmecophile interactions. We assume that the host resemblance in anatomy and behavior, as found in Wasmannian mimics and various other myrmecophile taxa [2, 3, 8, 116–118], supplements chemical mimicry in deceiving host ants, so that ants mistake intruders for nestmates. More than 100 years after Wasmann’s tactile mimicry hypothesis, the importance of tactile cues in ant nestmate recognition and in tactile mimicry of myrmecophiles is still largely unexplored. Anatomical and chemical mimicry of host ants in a variety of unrelated myrmecophiles (e.g., [3, 8, 18, 31]) suggests that the ants’ tactile and odor inspection via the antennae is a prime selective agent driving some of the most integrated myrmecophiles to mimic both, host body shape and host CHC profile (see also discussion in [4]).

### Specialization - host specificity trade-off

The evolution of host-specialization in parasites and parasitoids is considered to be a trade-off [10]. Specialization on a single host species is supposed to increase a parasite’s fitness on that particular host, but it comes at the cost of a high dependency on a single host species [10]. For example, a recent community study of host specificity in phorid parasitoids infecting leaf-cutter ants revealed high degrees of host specificity with 13 out of 20 species exploiting only a single host species [13]. We have compared the level of specialization in terms of social and chemical integration between army ant-associated beetle specialists and beetle generalists. Our results support the hypothesis that the evolution of host-specialization in parasites is a trade-off between the range of potential host species and the level of specialization (see also [92]). However, the present study only focused on two beetle groups, and species within the groups were phylogenetically closely related. To test whether this pattern universally holds for ant-parasite communities, future studies need to include a greater diversity of species including phylogenetically more distantly-related parasites.

## Additional files

Additional file 1: **Table S1.** Colony collection data (downloadable on the journal’s webpage; not included here). (XLSX 13 kb)

Additional file 2: **Table S2.** Specimen and collection information including GenBank accession numbers (downloadable on the journal’s webpage; not included here). (XLSX 36 kb)

Additional file 3: **Figure S1.** Assessment of species boundaries in *Eciton*-associated Wasmannian mimics via nuclear loci. **Figure S2.** Dry weight measurements of ants and parasites. **Figure S3.** Relationship of dry weight and CHC amount. **Figure S4.** Distribution of pairwise p-distances. **Figure S5.** Morphological distinction of studied *Ecitomorpha* species. **Table S3.** Re-assignment of samples to groups using a DAPC analysis. (PDF 4155 kb)
