# Supplementary material for: Chemical and behavioral integration of army ant-associated rove beetles – a comparison between specialists and generalists
Source: Front Zool. 2018 Mar 16;15:8. doi: 10.1186/s12983-018-0249-x (PMC5857133; doi:10.1186/s12983-018-0249-x)
Supplement: Supplementary file 4 — Figure S1. Assessment of species boundaries in Eciton-associated Wasmannian mimics via nuclear loci. Figure S2. Dry weight measurements of ants and parasites. Figure S3. Relationship of dry weight and CHC amount. Figure S4. Distribution of pairwise p-distances. Figure S5. Morphological distinction of studied Ecitomorpha species. Table S3. Re-assignment of samples to groups using a DAPC analysis. (PDF 4155 kb) [file 12983_2018_249_MOESM3_ESM.pdf]

Supplemental Material

Chemical and behavioral integration of army ant-associated rove beetles – a comparison between specialists and generalists

Christoph von Beeren[1,2*], Adrian Brückner[1], Munetoshi Maruyama[3], Griffin Burke[2,4], Jana Wieschollek[1], and Daniel JC Kronauer[2]

[1]*Ecological Networks, Department of Biology, Technische Universität Darmstadt, 64287 Darmstadt, Germany*

[2]*Laboratory of Social Evolution and Behavior, The Rockefeller University, New York, NY 10065, USA*

[3]*The Kyushu University Museum, Hakozaki 6-10-1, Fukuoka, 812-8581 Japan*

[4]*Bard College, Annandale-on-Hudson, NY 12504, USA.*

Content:

Figure S1. Assessment of species boundaries in *Eciton*-associated Wasmannian mimics via nuclear loci.

Figure S2. Dry weight measurements of ants and parasites.

Figure S3. Relationship of dry weight and CHC amount.

Figure S4. Distribution of pairwise p-distances.

Figure S5. Morphological distinction of studied *Ecitomorpha* species.

Table S1. Colony collection data (downloadable on the journal's webpage; not included here).

Table S2. Specimen and collection information including GenBank accession numbers (downloadable on the journal's webpage; not included here).

Table S3. Re-assignment of samples to groups using a DAPC analysis.

Figure S1. Assessment of species boundaries in *Eciton*-associated Wasmannian mimics via nuclear loci. Neighbor-joining trees based on Tamura-Nei distances (scale bars) were used as clustering analyses to detect possible species boundaries. Bootstrap support values are given at major branches. The phylogenetic outgroup (*Ecitoglossa* sp. GenBank accession numbers; *CAD*: MG191588; *Wg*: MG547401) has been excluded from the figure for better visibility (indicated by dashed lines). Portions of the nuclear genes *CAD* and *Wg* recovered the same genetic clusters as our analysis of *COI* (Fig. 2a). The following primer pairs were used. Asterisks indicate those primer pairs that most constantly amplified the respective PCR product. Specimen images are not to scale.

<u>*Wg* forward primers:</u>

Wg550F [1],

*Wg578F_tetra (TGCACGGTGAAGACCTGCTGGATG),

Wg578F_Tetr_Ecbi (TGCACGGTGAAGACSTGCTGGATG);

<u>*Wg* reverse primers:</u>

*WgAbrZ and WgAbr (both [1]),

<u>CAD forward primers:</u>

CD630F and CD667F (both [1])

*CD667F_Ecito_cl.2 (AACACAGATGAATTGAAATCGCTCGC)

<u>*CAD* reverse primers:</u>

*CD851R [1]

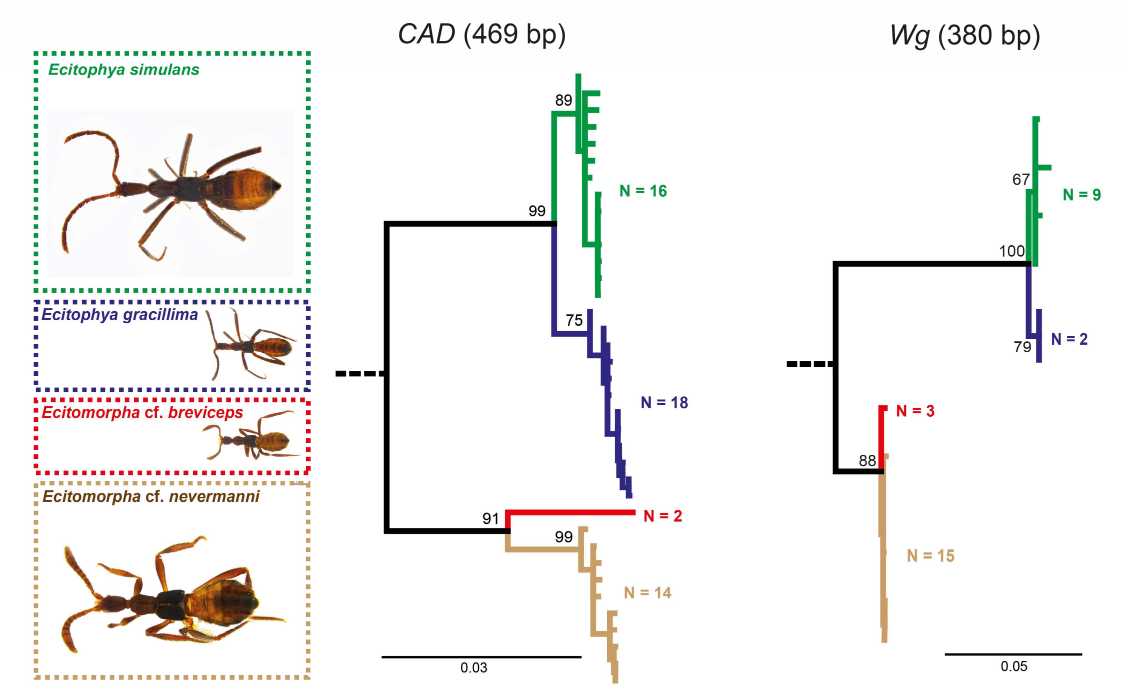

Figure S2. Dry weight measurements of ants and parasites. Dry weight of (a) ant workers from different castes, (b) parasites, and (c) females and males of *Ecitophya simulans*. Boxplots were created with RStudio (vers. 1.0.143) using the default settings for the command 'boxplot()'. Dots, i.e. individual data points, were added using the package 'beeswarm' (vers. 0.2.3). Bottom and top of boxes represent the 25th and 75th percentile, respectively. Median is shown by a thickened black line. Whiskers extend to 1.5 times the length of the boxes. Abbreviations: EB = *Eciton burchellii foreli*; EH = *Eciton hamatum*; E. bre. = *Ecitomorpha* cf. *breviceps*; E. nev. = *Ecitomorpha* cf. *nevermanni*; E. sim. = *Ecitophya simulans*; T. las. = *Tetradonia laselvensis*; T. mar. = *Tetradonia* cf. *marginalis*.

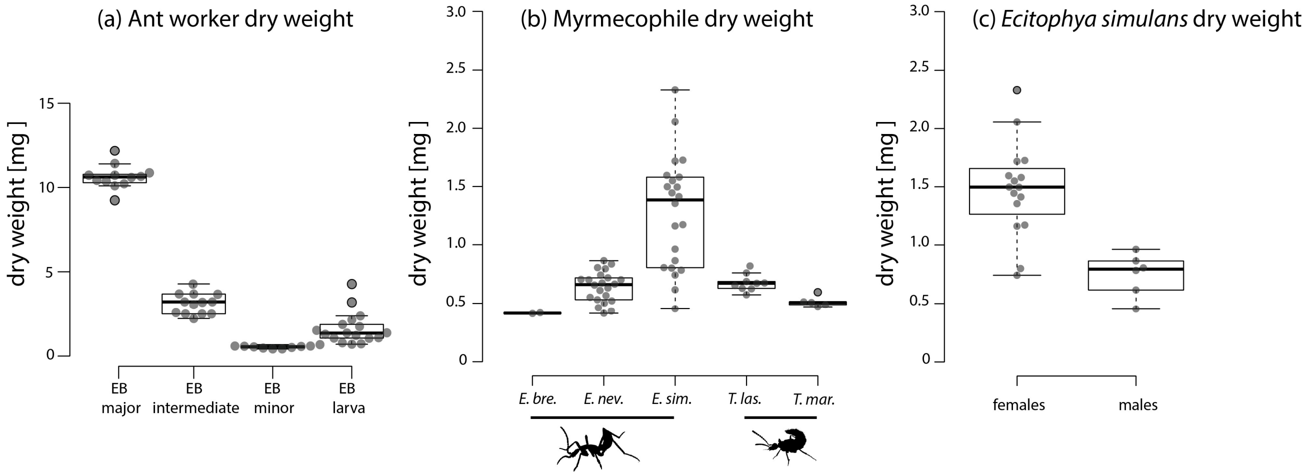

Figure S3. Relationship between specimen dry weight and CHC amount. Linear models with dry weight as independent and amount of CHC as dependent variable were calculated separately for the different categories 'ants', 'specialists', and 'generalists'. Both variables were log-transformed (log (dry weight + 1) and log (amount CHC + 1)) to follow a log-normal distribution. CHC amount is plotted against animal dry weight for *Eciton burchellii foreli* workers (blue circles, N =28), beetle specialists (green circles, N = 45) and beetle generalists (pink circles, N = 14). We found a linear relationship between the two variables for ants (linear model: F-value: 169.1, P < 0.001), but not for specialists (linear model: F-value: 0.071, P = 0.792) or generalists (linear model: F-value: 0.029, P = 0.869). Solid blue line shows the linear line of best fit for ant data and dashed blue line the 95% confidence interval. Ant larvae were excluded from linear regression analyses as they carried only very small amounts of CHCs.

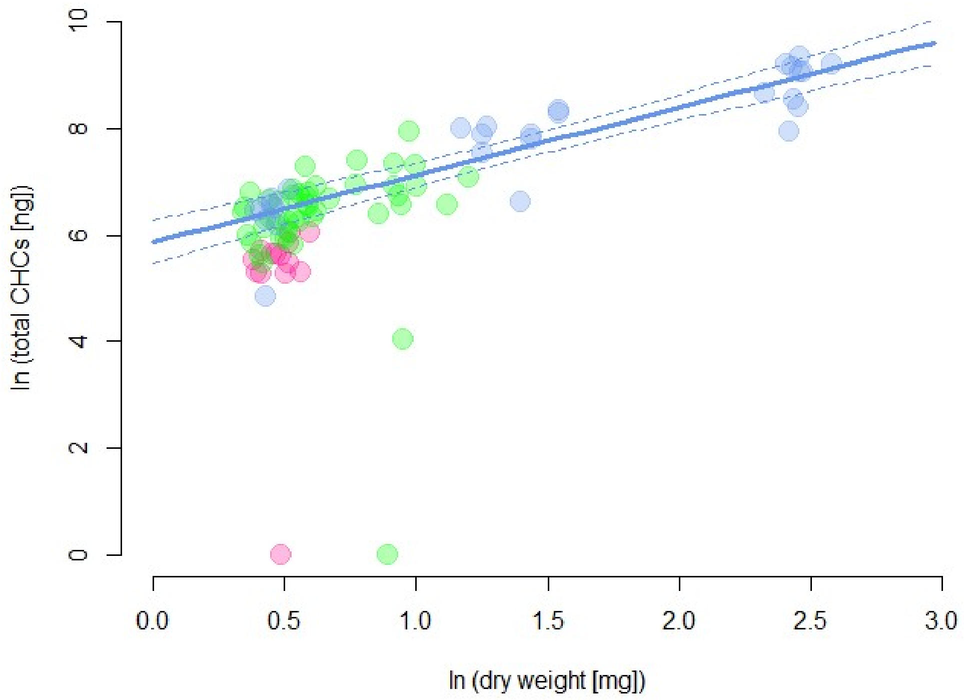

Figure S4. Distribution of pairwise p-distances. Histogram showing the intra- and interspecific p-distances in pairwise comparisons of *COI* sequences among the four species *Ecitophya simulans*, *Ecitophya gracillima*, *Ecitomorpha* cf. *breviceps*, and *Ecitomorpha* cf. *nevermanni*. P-distances give the proportion of bases that differ in pairwise-comparisons. The red arrow indicates a 'barcode gap', i.e. a gap between smallest interspecific and largest intraspecific p-distances.

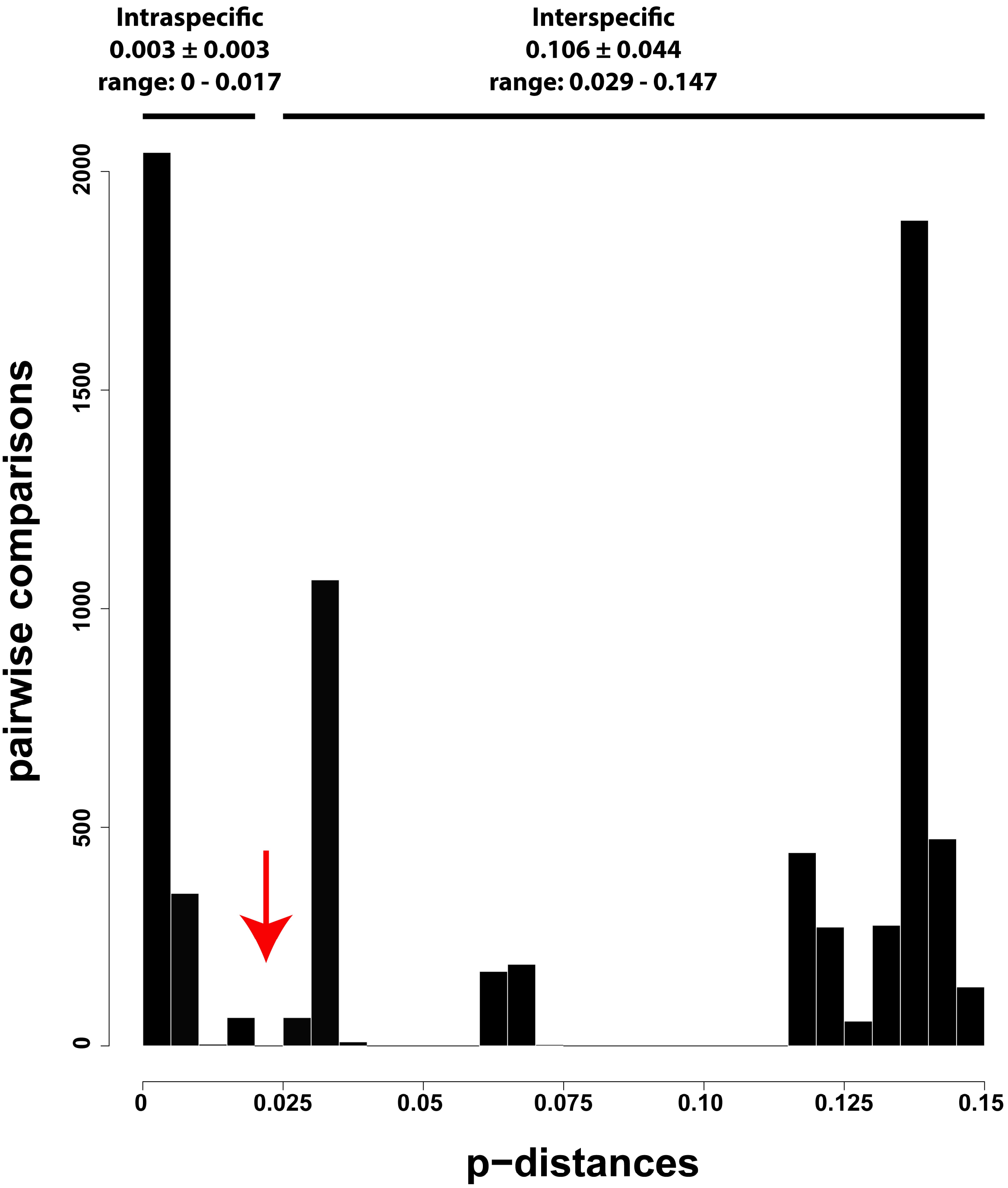

Figure S5. Morphological identification of *Ecitomorpha* species. (a, b) Specimen of *Ecitomorpha* cf. *breviceps* (left) and specimen of *Ecitomorpha* cf. *nevermanni* (right). The *Ecitomorpha* specimens studied here fit well to the species descriptions of *E. breviceps* and *E. nevermanni* [2,3]. The following morphological features allowed us to reliably distinguish the two *Ecitomorpha* species studied here. For further anatomical details of the species see the original species descriptions [2,3]. (a, b) Head of *E.* cf. *breviceps* is roundish, not much longer than wide. In contrast, head of *E.* cf. *nevermanni* is elongated, twice as long as wide. (a) Last antennal segments are as long as wide in *E.* cf. *breviceps* (white arrows), but they are longer than wide in *E.* cf. *nevermanni* (white arrows). (b) Pronotum with a narrow deep incision opening up anteriorly in *E.* cf. *nevermanni* (white arrows), but not in *E.* cf. *breviceps* (white arrows).

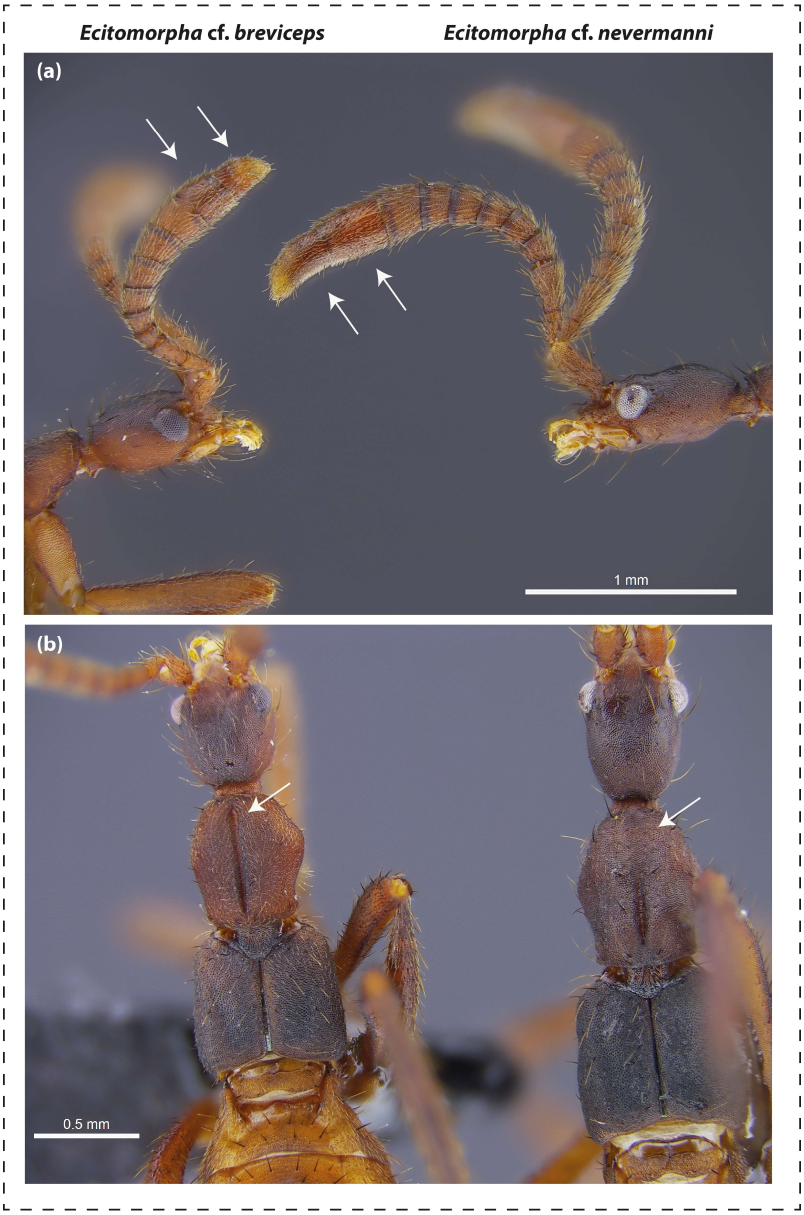

		E. burchellii foreli				beetles		sum
		larva	minor	intermediate	major	Specialists	Generalists	
E. burchellii foreli	**larva**	-	-	-	-	8	-	8
	**minor**	-	1	-	-	9	-	10
	**intermediate**	-	-	-	-	8	-	8
	**major**	-	-	-	10	-	-	10
**beetles**	**Specialists**	2	-	-	-	41	1	44
	**Generalists**	-	-	-	-	2	11	13

Table S3. Re-assignment of samples to groups using a discriminant analysis of principal components (DAPC). A DAPC classified samples into *a priori* defined groups. CHC composition of most larvae, minors, and intermediate workers were assigned to the category 'Specialist'. The CHC profiles of Wasmannian mimics (specialists) and ant workers (except majors) could thus not be distinguished via a DAPC.

References

1. Wild AL, Maddison DR. Evaluating nuclear protein-coding genes for phylogenetic utility in beetles. Mol Phylogenet Evol. 2008;48:877–91.

2. Reichensperger A. Ecitophilen aus Costa Rica (II), Brasilien und Peru (Staph. Hist. Clavig.). Rev Entomol. 1933;3:179–94.

3. Reichensperger A. Beitrag zur Kenntnis der Myrmekophilenfauna Brasiliens und Costa Ricas III.(Col. Staphyl. Hist.). Arb morph taxon Ent Berlin-Dahlem. 1935;2:188–218.

4. Kistner DH, Jacobson HR. Cladistic analysis and taxonomic revision of the ecitophilous tribe Ecitocharini with studies of their behavior and evolution (Coleoptera, Staphylinidae, Aleocharinae). Sociobiology. 1990;17:333–480.
